# The association between food insulin index and odds of non-alcoholic fatty liver disease (NAFLD) in adults: a case-control study 

**Published:** 2021

**Authors:** Somaye Fatahi, Mohammad Hassan Sohouli, Appaji Rayi, Farshad Teymoori, Farzad Shidfar

**Affiliations:** 1 *Student Research Committee, Iran University of Medical Sciences, Tehran, Iran *; 2 *Department of Nutrition, School of Public Health, Iran University of Medical Sciences, Tehran, Iran*; 3 *Department of Neurology, Charleston Area Medical Center, Charleston, WV, USA*; 4 *Nutrition and Endocrine Research Center, Research Institute for Endocrine Sciences, Shahid Beheshti University of Medical Sciences, Tehran, Iran *

**Keywords:** Insulin, Biochemical processes, Obesity, Chronic diseases, Non-alcoholic fatty liver disease

## Abstract

**Aim:**

This research aimed to study the association of food insulin index and biochemical parameters with the **odds** of developing NAFLD in adult Iranians.

**Background:**

Hyperinsulinemia may play an important role in the development of non-alcoholic fatty liver disease (NAFLD) because of the relationship between insulin response and body fat accumulation.

**Methods:**

A case-control study of 169 NAFLD patients and 200 healthy adults aged 18-55 years was conducted. Dietary data was collected using a validated 168-item quantitative food frequency questionnaire (FFQ). Food insulin index (FII) was calculated by dividing the total insulin load by total energy intake (kcal/day). Total insulin load (ILoverall) was also calculated using a standard formula.

**Results:**

Mean participant age was 43.9 ± 5.9 years. Patients with NAFLD were significantly associated with higher body mass index, levels of liver enzymes, triglyceride, low density lipoprotein-cholesterol (LDL), total cholesterol, and fasting blood sugar (FBS) compared to the healthy subjects (*p* < 0.05). The highest tertiles of FII were associated with higher odds of NAFLD (OR=1.4, 95% CI: 0.88-2.48, *p* for trend <0.001) and obesity (OR=2.33, 95% CI: 0.97-5.75) compared to the lowest tertiles. Potential confounders for the association were controlled.

**Conclusion:**

This study found that adherence to a diet with high FII was associated with greater odds of NAFLD and overweight or obesity. Additional studies are required to better understand this association.

## Introduction

 Nonalcoholic fatty liver disease (NAFLD) is a result of fat accumulation over 5% in the liver in the absence of any known risk factors like viral infection, drug abuse, immune or metabolic disorders (1, 2). The prevalence of NAFLD has been increasing and more than 25% of the world's population is affected (3). Its prevalence among the adult population in Iran is approximately 20% (4). Growing evidence confirms that conditions like obesity, type 2 diabetes mellitus (T2DM) (5), and metabolic syndrome are increasingly associated with NAFLD (6). There is significant concern about the potential of NAFLD progressing to cirrhosis, liver failure, and hepatocellular carcinoma (7). Moreover, NAFLD increases the risk of mortality in patients with cardiovascular and metabolic disorders (5). 

As there are no clear pharmacological treatments for this condition, the primary recommendation in most cases is lifestyle modification and related interventions (8). Among these, a healthy diet independent of weight or physical activity in early stages can certainly modify the disease progression (9). Poor dietary habits result in hyperinsulinemia and insulin resistance, which play a crucial role in the development of NAFLD by interfering with energy balance pathways (10). Postprandial insulin secretion in response to glucose, fructose, certain amino acids, and fatty acids that have a high insulinogenic potential plays an important role in the progression of chronic disease (11). Therefore, the dietary insulinemic potential of diet may be considered as a mediator factor for NAFLD independent of other factors.

The Food Insulin Index (FII) is a new food ranking algorithm based on the body's insulin response to the use of Isoenergetic Reference Food in healthy individuals (12). This algorithm expresses that any dietary components and metabolic interactions can have a significant effect on insulin demand with sufficient energy density. Previous studies have shown that this algorithm predicts the rate of insulin response more accurately than carbohydrate counts (11, 13). FII compared to carbohydrate counts in type 1 diabetic patients has been shown to improve postprandial glycemia without increasing the risk of hypoglycemia (14). In single foods, FII can predict insulin response better and more accurately than carbohydrate count and glycemic index (13). Recently, several studies have indicated a potential link between FII and reduced risk of chronic diseases such as cancer (15), metabolic syndrome (11), and overweight or obesity as well (16). 

To the best of our knowledge, the association between FII and odds of NAFLD as well as FII and liver function have not been investigated. Thus, the present study aimed to investigate the relationship between FII in patients with NAFLD in order to understand the diet-based insulin response. 

## Methods

***Participants***

The current study was conducted after obtaining approval from the Research Council and Ethics Committee at Iran University of Medical Sciences, Tehran, Iran. This hospital-based case-control study was performed in Hazrat Rasoul Hospital, Tehran, Iran, in the years 2018–2019. In this study, 169 consecutive patients with NAFLD (randomly selected) diagnosed by a gastroenterologist were included in the case group, and 200 individuals without a history of NAFLD were recruited from the same hospital for the control group. Two dietitians (Mh. S. and S. F.) monitored the sampling of patients. NAFLD was diagnosed based on the chronic elevation of liver enzymes, absence of alcohol consumption, ultrasonography of the liver showing evidence of NAFLD based on echogenicity and visualization of vasculature, and parenchyma and diaphragm (Grades II and III). Patients with liver disease secondary to other known etiologies and a history of diabetes, CVDs, myocardial infarctions/strokes, cancers, viral hepatitis, Wilson’s disease, or autoimmune disorders of the liver were excluded. Pregnant and lactating women and subjects with arbitrary special diets were also excluded from the study. NAFLD patients were newly diagnosed and not treated with any medication or surgery before the study. Healthy subjects were considered as a control group and were included based on laboratory tests and liver ultrasonography (not suffering from stages of hepatic steatosis). The control group was selected from patients referred to other wards of the hospital, such as ophthalmology, orthopedics, maxillofacial surgery, and ear, nose, and throat wards, who were not diagnosed with NAFLD and had no history of alcohol consumption overall or had a history of alcohol consumption less than 10 mg/d in women and less than 20 mg/d in men. Case and control group subjects were age and sex-matched. Demographic data was collected through a general information questionnaire. At the beginning of the study, the levels of liver enzymes were recorded using the patient's existing medical record or newly obtained at a doctor’s appointment if unknown. In this study, the nutritionists performed the interviews, and thus the responder rate to the survey was complete. Furthermore, written informed consent was obtained from all participants. The General Practice Physical Activity Questionnaire (GPPAQ), a simple questionnaire reflecting an individual’s current physical activity, was used to assess the physical activity of participants (17). The study protocol was ethically approved by the Regional Bioethics Committee of Iran University of Medical Sciences (NO: IR.IUMS.REC.1398.448).

**Anthropometric assessment**

Anthropometric measurements were carried out by a trained dietician. Weight was measured with minimum clothes and no shoes using a standard SECA 700 digital scale (SECA, Germany) and recorded to the nearest 100 g. Height was measured in a standing relaxed shoulder position with no shoes to the nearest 0.5 cm using mounted tape (SECA Stadiometer, Germany). Body mass index (BMI) was calculated as weight (kg) divided by height in square meters (m^2^). 

**Measurement of biomarkers**

After a 12-hour period of fasting, 10 ml of fasting blood was taken between 7 and 10 am from all participants. Blood samples were centrifuged for 10 min at 500 g and at 4 °C within 30–45 min of collection. The serums were shed in clean microbeads and stored at -80 °C until testing. Then, triglyceride, cholesterol (C), high density-lipoprotein (HDL)-C, low-density lipoprotein (LDL)-C, and glucose concentrations were measured by enzymatic methods using commercial kits (Pars Azmoon, Tehran, Iran). Alanine aminotransferase (ALT) and aspartate aminotransferase (AST) were determined by commercially available enzymatic reagents (Pars Azmoon, Tehran, Iran) on auto-analysis (BT-3000).

**Dietary assessment**

Trained dietitians administered usual interviews to chart food intake. Validated semi-quantitative food frequency questionnaires (FFQ) with 168 food items were used to assess dietary intakes (18). For each food item, a standard unit or portion size was specified and participants were asked how often, on average, during the previous year they had consumed that amount. The participants reported the consumption frequency of each food item per day, week, month, or year. Responses to individual food items were converted to average daily intake of each food item. The nutrient and energy content of the foods were analyzed using Nutritionist 4 software (First Databank Inc., Hearst Corp., San Bruno, CA) modified for Iranian foods.

**Calculation of food insulin index and glycemic index**

Food insulin index (FII) refers to the incremental insulin area under the curve over 2 h in response to the consumption of a 1000-kJ portion of the test food divided by the area under the curve after ingestion of a 1000-kJ portion of the reference food. Glycemic index (GI) refers to the postprandial glucose response to a food compared with an equal amount of carbohydrate (50 g) from a reference food, usually glucose or white bread (19). Glucose was used as a reference food to calculate the GI variable in the current study. The overall dietary GI was calculated by adding the products of the carbohydrate content of each food, multiplying this sum by its GI, multiplying this number by the average number of servings consumed per week, and then dividing the result by the total weekly intake of available carbohydrates. The total dietary glycemic load (GL) of each food was calculated by multiplying the quantity of each food item consumed per day by the amount of carbohydrates contained in a specified serving size of the food and its corresponding GI value. Total GL was then estimated by summing the values for all carbohydrate-containing foods reported on the FFQ, whereas total GI was calculated by dividing the total dietary GL by the total available carbohydrate intake. The GI table developed for Iranian food items was used to calculate GL and GI (20). As the Iranian table of GI is incomplete, the international tables of GI and GL values were also used (21). The insulin index for each calorie-containing food was obtained from FFQ data published by Professor Jennie Brand-Miller of the University of Sydney, Australia. For each study participant, the total insulin load or the overall insulin load (ILoverall) over the past year for each calorie-reported food in the FFQ was determined by calculating its index FII, the calorie content of that food (kcal per portion of that nutrient intake), and its frequency of use (portion/daily) and then the sum of the amounts (22). The overall insulin index (IIoverall) was also calculated by dividing ILoverall by total energy intake (kcal/day).

${\mathrm{II}}_{\mathrm{ave}}=\frac{\sum_{a=1}^{n}\left({\mathrm{II}}_{a}\times {\mathrm{Energy}}_{a}\times {\mathrm{Frrequency}}_{a}\right)}{\sum_{a=1}^{n}\left({\mathrm{Energy}}_{a}\times {\mathrm{Frrequency}}_{a}\right)}$

${\mathrm{II}}_{\mathrm{ave}}=\sum_{a=1}^{n}{\mathrm{II}}_{a}\times {\mathrm{Energy}}_{a}\times {\mathrm{Frrequency}}_{a}$

**Statistical analyses**

The normality of variables was evaluated by Kolmogorov-Smirnov and histogram tests. For the variables that did not have a normal distribution, the logarithmic equivalent (Ln transformation) of that variable was used in the analysis. The independent sample T test was used to compare the mean of food insulin index as well as the general characteristics and biochemical parameters among the case and control groups. To assess the relationship between FII and NAFLD in an adjusted model, multiple logistic regressions were used. Multiple logistic regressions adjusted for confounders were used to evaluate the association between FII and anthropometric and biochemical variables. The data is presented as mean ± standard deviation and odds ratio with 95% confidence interval. A value of *p* < 0.05 was considered significant. All statistical analyses were done using SPSS software (version 19.0; SPSS Inc., Chicago IL).

## Results

The mean age and BMI of the study participants were 43.9 ± 5.9 years and 30.5 ± 2.6, respectively. The anthropometric and biochemical indices among case (NAFLD patients) and control groups are described in Table 1. No significant differences were seen between the case and control groups except that subjects with NAFLD had a higher BMI and weight (*p* <0.001), higher levels of FBS, TG, LDL, TC, and ALT compared with the control group (*p* <0.05), and lower HDL and physical activity levels compared with the control group (*p* <0.05). 

No significant differences were found between the NAFLD and control subjects based on the glycemic load (see Table 2); however, the dietary glycemic index was significantly higher (*p* <0.001). Subjects with NAFLD also had a higher intake of energy, fats, carbohydrates, saturated fatty acids, fructose, refined grains, high-fat dairy products, red and processed meats and a lower intake of total fiber, fruit, whole grains, and vegetables compared with the control subjects. There were no differences in the consumption of other dietary products between the groups. 

The odds ratios (ORs) and 95% confidence intervals (Cis) for NAFLD and obesity in the tertiles of FII are described in Table 3. Compared to participants in the lowest tertile of FII, those in the highest tertile included significantly lower ORs for NAFLD after adjustments for potential confounders (OR and 95% CI =2.87 (1.18 – 6.96), *p-*value for trend = 0.017). Despite a significantly increasing trend of ORs across tertiles of FII for high LDL, HDL, and FBS levels, a similar trend was not seen in the triglyceride and liver enzyme levels. In addition, the highest versus the lowest tertiles of FII showed higher **odds** of overweight or obesity (OR and 95% CI = 3.61 (1.87–6.97), *p-*value for trend <0.001) in the final adjusted model for potential confounders including age, sex, and BMI.

**Table 1:** Anthropometric and biochemical parameters among case and control groups

Variables	Case (n=169)	Control (n=200)	p-value
Women, %(No)	58.7(125)	51.3(88)	0.06 ^b^
Age, years	42.75±5.9^*^	45.13±5.9	0.06^ c^
Weight, kg	94.44 ± 15.6	74.14 ± 12.9	<0.001^ c^
Physical Activity (Met.min/wk)	984.5±1241.5	1563.2 ± 1216.7	<0.001 ^c^
BMI, kg/m^2^	33.19± 3.1	27.95 ± 2.1	<0.001^ c^
FBS, mg/dl	109.29 ± 7.6	92.21 ± 6.7	<0.001^ c^
Total cholesterol, mg/dl	184.79± 42.1	182.85 ± 40.1	<0.001^ c^
Triglycerides, mg/dl	180.40± 14.3	130.99 ±12.2	0.001^ c^
HDL, mg/dl	41.26 ±15.1	48.5 ± 17.3	0.017^ c^
LDL, mg/dl	121.17 ± 23.4	109.14 ± 13.0	<0.001^ c^
ALT, mg/dl	58.50 ±24.1	20.53 ±13.01	<0.001^ c^
AST, mg/dl	34.88 ±17.2	23.76 ± 9.6	0.17^ c^
Glycemic load	266.16 ± 85.9	264.07± 94.8	0.82
Dietary glycemic index	56.76± 5.08	54.29 ± 5.34	<0.001


^* ^mean ± SD^ a^ Non-alcoholic Fatty Liver Patients; ^b^p-values resulted from chi square; ^c ^p-values resulted from student t-test

**Table 2:** Dietary Intake and Dietary Glycemic Index among Casea and Control Groups

Variables	Case (n=169)	Control (n=200)	p-value^b^
Energy, kcal	2741.8± 819.89^*^	2427.67 ± 798.09	0.04
Macronutrients			
Protein, gr	97.33 ± 34.37	104.47 ± 39.61	0.06
Fat, gr	103.44 ± 40.11	86.27 ±32.58	<0.001
Saturated fatty acid	32.13±6.35	26.87±6.42	0.006
Carbohydrate, gr	408.06 ± 148.95	378.44 ± 112.63	0.03
Fiber, gr	46.36 ± 18.25	47.58 ± 19.76	0.5
Fruits	249.38±190.29	368.03±192.15	<0.001
Vegetables	269.36± 183.63	339.31±185.25	<0.001
Whole grain	121.54±80.71	134.44±79.91	<0.001
Refined grain	420.41±140.35	368.87±141.65	<0.001
Fructose	25.95±12.19	23.52 ± 10.33	0.03
Legumes (g/d)	18.8±1.5	17.4±0.7	0.367
Fish (g/d)	9.3 ±0.5	9.4 ±0.4	0.897
Red and organ meats (g/d)	26.1±1.6	22.0±0.9	0.023
Processed meats (g/d)	8.8 ±9.4	2.6 ±4.3	<0.001
High-fat dairy (g/d)	129.8±7.6	55.2±3.1	<0.001
Low-fat dairy (g/d)	229.9 ±11.5	224.2 ±7.8	0.679
Coffee and tea (g/d)	619.6 ±38.4	613.6 ±25.2	0.894
Nuts (g/d)	6.3 ±0.4	6.3 ±0.5	0.909

^* ^mean ± SD^ a^ Non-alcoholic Fatty Liver Patients; ^b ^*p-*values resulted from student t-test

**Table 3:** Association between Tertiles Dietary Insulin Index with Risk of NAFLD and Biochemical Parameters

Variable (No case/No control)	Tertiles of food Insulin Index			P trend
	T1(30/67)	T2(57/67)	T3(82/66)	
NAFLD^a^				
Model 1*	1.00 (Ref)	2.8(1.6-4.83)	1.4(0.88-2.48)	<0.001
Model 2†	1.00 (Ref)	1.45(0.72 - 2.94)	2.87 (1.18 – 6.96)	0.017
Overweight or obesity^b^				
Model 1*	1.00 (Ref)	0.69 (0.23- 2.58)	2.33 (0.97-5.75)	0.04
Model 2‡	1.00 (Ref)	1.72 (0.85–3.50)	3.61 (1.87–6.97)	< 0.001
High FBS level^a^	1.00 (Ref)	1.18 (0.66-2.09)	1.98 (1.13- 3.49)	0.016
High LDL level^a^	1.00 (Ref)	1.87(0.92-3.8)	2.77(1.39-5.50)	0.004
High TG level^a^	1.00 (Ref)	1.62(0.85- 3.08)	2.4(1.21-4.5)	0.005
High HDL level^a^	1.00 (Ref)	1. 90(1.02-3.81)	1.81(0.96-3.52)	0.08
High ALT level^a^	1.00 (Ref)	2.26(0.69-5.1)	1.68(0.61-3.1)	0.04
High AST level^a^	1.00 (Ref)	2.9(0.9-3.1)	1.73(0.51-2.13)	0.03

* Model 1: adjusted for age and sex; † Model 2: adjusted for model 1 and BMI, physical activity, biochemical parameters, energy intake, fructose, fat intake, refined grains; ‡ Model 2: adjusted for model 1 and physical activity, biochemical parameters, and energy intake; fasting blood sugar (FBS), high density lipoprotein (HDL), low density lipoprotein (LDL), alanine aminotransferase (ALT), aspartate aminotransferase (AST)

## Discussion

The present case-control study investigated the relationship between FII and the odds of NAFLD. It was found that FII and the glycemic index have a strong positive association with NAFLD and obesity odds among adult patients in Iran. Moreover, FII was significantly associated with lower HDL and higher liver enzyme, triglycerides, LDL, and FBS levels.

According to our research, no study has yet investigated the association between FII and NAFLD. Importantly, recent research has recognized FII as a predictor of the risk of chronic diseases (23). Several studies have suggested that FII leads to increased risk of diabetes, CVD, and cancer (14, 24-27). All these conditions share common metabolic parameters with NAFLD. In another recent cohort study, after controlling for potential confounders, women in the highest quartile of FII had 41% greater odds of having metabolic syndrome compared with those in the lowest quartile. This finding is completely consistent with the current results. In addition, findings from both the Nurses’ Health Study and the Health Professionals Follow-up Study showed that adherence to a diet with a high insulin index and load was associated with increased serum levels of TG, and FII was inversely associated with serum HDL-C concentrations (26).

Several possible mechanisms have been considered to explain the association between FII and the risk of NAFLD. Studies have suggested that dietary insulin load and FII could be considered as independent dietary risk factors for the development of insulin resistance that contributes to its pathogenesis NAFLD (28, 29). Insulin resistance in muscle and adipose tissue leads to metabolic abnormalities such as inappropriate release of fatty acids through dysregulated lipolysis and disorders of glucose metabolism that further contribute to impaired insulin signaling. This change increases the free glucose in the blood, which is converted to fat that eventually accumulates in the liver, increasing the risk of NAFLD. Furthermore, disorders of insulin release increase the release of adiponectin, IL-6, and other peptides by adipose tissue that causes inflammatory effects in the liver (13, 29, 30). Overall, hyperinsulinemia and insulin resistance play an important role in the development of NAFLD. Moreover, increased dietary glycaemia, intake of foods with high insulin response, as well as hyperlipidemia through increased oxidative stress or reactive oxygen species (ROS) in obese or diabetic individuals lead to liver fibrosis and ultimately increase the chances of developing nonalcoholic fatty liver (31-33).

In the present study, patients with NAFLD consumed lower amounts of vegetables and whole grains compared to refined grains, fructose, and saturated fatty acids that could typically lead to the risk of developing NAFLD and other biochemical disorders of impaired lipid profile and glucose metabolism; however, they had a lower intake of fiber, which is usually associated with a higher body mass index, fat accumulation, and higher levels of serum lipids (34). 

In the present study, a significant positive relationship was observed between FII and the **odds** of overweight or obesity in the highest compared with the lowest tertiles of FII. In a German cohort study in young adults, a higher FII during puberty was associated with a higher percent of body fat (35). Moreover, Katharina et al. reported a higher FII in obese individuals (26). In contrast, another cohort study did not find any significant association between FII and the risk of abdominal obesity (36). This discrepancy might be explained by the different tools used for dietary assessment, subjects’ age ranges, considering different variables as confounders, and methods for calculating FII values. In addition, different food processing and cooking methods in different cultures could be another reason for the discrepancy. The potential mechanisms of the effect of FII on obesity or overweight are unknown. A high FII may be due to the stimulation of insulin secretion and, thus, an increase in fat synthesis and carbohydrate oxidation, leading to obesity or overweight (37). Some studies have suggested that high FII foods are rapidly digested, absorbed, and converted to glucose. This can cause a rapid fall in glucose excursion, which is associated with reduced satiety and excessive caloric intake (38, 39). It also appears to be due to the low insulinemic dietary pattern found in whole fruits, vegetables, and leafy green vegetables (containing nutrients rich in fiber, calcium, magnesium, and potassium), that is less common in red meats, processed meats, sugars, sugary drinks, and chicken (40). On the other hand, various studies have shown that vegans have better regulation of triglyceride-rich lipoprotein metabolism, because they are more efficient at eliminating potentially atherogenic residues. This may justify the reduction in chylomicron residues when using low insulinemic dietary products (26, 29).

There are a few limitations that need consideration while interpreting the results of the current study. Firstly, like all case–control studies, no causal relationship could be determined between FII and NAFLD. Secondly, using the 168-item FFQ questionnaire might contribute to a recall bias from tired participants, which might have been partially addressed by a trained interviewer. Thirdly, some properties of foods, like growing conditions, cultivation methods, storage, processing, and cooking conditions, and assay methods, might affect the antioxidant contents of the foods, and that needs to be considered as well (41). Nevertheless, this is the first study to examine the relationship between FII and NAFLD in a case control design, based on which further research needs to be undertaken. Likewise, a wide range of variables was adjusted and a validated questionnaire was used in an attempt to eliminate the effects of confounders in the best way possible. 

In conclusion, a high FII diet may be associated with higher odds of developing NAFLD and obesity or overweight due to its positive association with plasma triglycerides, LDL, and FBS levels. Moreover, there is a negative association between FII and HDL and liver enzyme levels in NAFLD, suggesting that FII has long-term metabolic relevance, especially in individuals with a higher grade of insulin resistance. More studies are required to evaluate the association between FII and NAFLD in a larger population and to address any potential gender-based differences.
